# Coagulation Factor XII Is an Antibacterial Protein That Acts Against Bacterial Infection via Its Heavy Chain

**DOI:** 10.3390/ijms26136009

**Published:** 2025-06-23

**Authors:** Junnan Liu, Diyue Wang, Sirui Pan, Xu Song

**Affiliations:** Center for Functional Genomics and Bioinformatics, Key Laboratory of Bio-Resource and Eco-Environment of Ministry of Education, College of Life Sciences, Sichuan University, Chengdu 610064, China; liujunnan@stu.scu.edu.cn (J.L.); 15982863026@163.com (D.W.); pan_n@foxmail.com (S.P.)

**Keywords:** coagulation factor XII, antibacterial activity, disseminated intravascular coagulation (DIC)

## Abstract

Coagulation factor XII (FXII), the initiator of the intrinsic coagulation pathway, is not involved in hemostasis but is associated with pathological thrombosis. Bacterial infections activate coagulation cascades, although the underlying mechanisms remain not fully understood. Here, we revealed that FXII exhibits antibacterial activity through its heavy chain (hFXII) against *Pseudomonas aeruginosa* (*P. aeruginosa*), a Gram-negative bacterium. We constructed an FXII-deficient (FXII^−/−^) mouse model and demonstrated that FXII plays a critical role in antibacterial functions. FXII and hFXII significantly reduced bacterial loads via intravenous injection, confirming their antibacterial activity in FXII^−/−^. To further investigate the pathophysiological implications of FXII in the *P. aeruginosa*-induced disseminated intravascular coagulation (DIC) mouse model, FXII and hFXII effectively reduced DIC-related bacterial infections, alleviated organ damage, and decreased fibrin deposition, consequently improving survival rates. This study indicates that FXII exhibits both in vitro and in vivo antibacterial activity, primarily mediated through its heavy chain. In thrombotic diseases triggered by Gram-negative bacterial infections, the antibacterial functions of FXII may influence the progression of the disease. These results not only redefine the critical role of the intrinsic coagulation pathway in innate immune defense but also provide novel insights into the prevention and treatment of severe infection-related diseases.

## 1. Introduction

The coagulation system is characterized by an intricate cascade of enzymatic reactions, encompassing intrinsic and extrinsic pathways that converge into common pathways. This complex interplay of reactions converts soluble fibrinogen into insoluble fibrin [1,2]. Factor XII (FXII) circulates in plasma as an inactive zymogen and serves as the initiator of the intrinsic coagulation pathway [3]. Its activation occurs upon contact with negatively charged surfaces, where FXII is activated to FXIIa, which is composed of a heavy chain (HC) and a light chain (LC) linked by disulfide bonds [4]. The heavy chain primarily mediates recognition and binding functions, while the light chain exhibits serine protease activity to activate the coagulation cascade [5]. The activation of FXII elicits two canonical physiological responses: initiating the intrinsic coagulation cascade and triggering the kallikrein–kinin system (KKS) to subsequently release the pro-inflammatory mediator bradykinin (BK) [6,7,8]. Severe deficiency of FXII does not result in bleeding disorders, but individuals are prone to thrombosis and inflammatory responses [9,10]. This indicates that the contact system is not involved in hemostasis but is more closely associated with innate immunity. Interfering with this pathway may protect against thrombosis without significant bleeding risks, emphasizing its emerging role in combating invasive bacterial infections [11,12,13].

The discovery of antibiotics liberated clinical medicine from the burden of infectious diseases [14]. However, the evolution and global dissemination of antibiotic resistance genes in pathogenic bacteria have rendered previously treatable infections as lethal threats, with an estimated 4.71 million deaths worldwide in 2021 attributable to antimicrobial resistance (AMR) [15,16]. Resistance is recognized as one of the top 10 global threats requiring urgent action, with drug resistance research on *Acinetobacter baumannii* (*A. baumannii*) and *Pseudomonas aeruginosa* (*P. aeruginosa*) identified as critical priorities by the World Health Organization (WHO) in its ranking of bacterial pathogens that lack effective antibiotic therapies [17,18]. In some cases, no viable antibiotics remain. Bacterial infections stimulate innate immune responses and activate the coagulation cascade, which is critical for maintaining intravascular homeostasis [19,20]. Excessive activation of coagulation systems by endotoxins leads to life-threatening disseminated intravascular coagulation (DIC) [21,22]. Severe bacterial intra-abdominal infections (IAIs) and bloodstream infections (BSIs) can lead to sepsis, whose high mortality rate poses a significant global health challenge [23,24].

Recent studies on the antibacterial mechanisms of FXII have highlighted the critical role of the intrinsic coagulation pathway in innate immunity [8]. However, most effects are mediated indirectly through the activation of the contact system and the subsequent stimulation of the immune response, while direct antibacterial mechanisms remain largely unexplored. As a key initiator, the activation of FXII further activates the plasma contact system, driving pro-inflammatory and pro-coagulant pathways, complement activation, and fibrinolysis [25,26]. Both coagulation and inflammatory processes contribute to the pathophysiological response to bacterial infection [27]. Upon activation by anionic substances released from bacterial surfaces or host injury, FXII initiates the KKS, generating kallikrein and bradykinin [28]. The complement and coagulation systems are interconnected plasma protein cascades that serve essential roles in host defense and hemostasis [29].

Our previous studies have identified the antibacterial activity of FVII, FIX, and FX. FVII exerts its antibacterial activity by hydrolyzing lipopolysaccharide (LPS), with epidemic growth factor (EGF)-like1 and EGF-like2 as the functional domains [30]. As an essential protein in the intrinsic coagulation pathway, the true role of FXII is still being further explored. We hypothesized that FXII may possess similar antibacterial properties, playing a pivotal role in innate immunity.

In this study, we analyzed the antibacterial function of FXII, investigated the impact of FXII deficiency in *P. aeruginosa* infection models, and evaluated the antibacterial function of FXII in a DIC mouse model. Our findings suggest that discovering the antibacterial activity of FXII, as the initiator of the intrinsic coagulation pathway, may reshape our understanding of FXII as a dual-function protein with antibacterial and coagulation activities. In infection-triggered thrombotic diseases, FXII may serve as a novel therapeutic target to balance antibacterial and coagulation functions, enabling effective bacterial elimination without excessive coagulation activation. This innovative perspective could offer new insights into the mechanisms of infection-induced thrombotic diseases and inform innovative strategies for their prevention and treatment.

## 2. Results

### 2.1. FXII Exhibits Antibacterial Activity In Vitro

**Figure 1:**
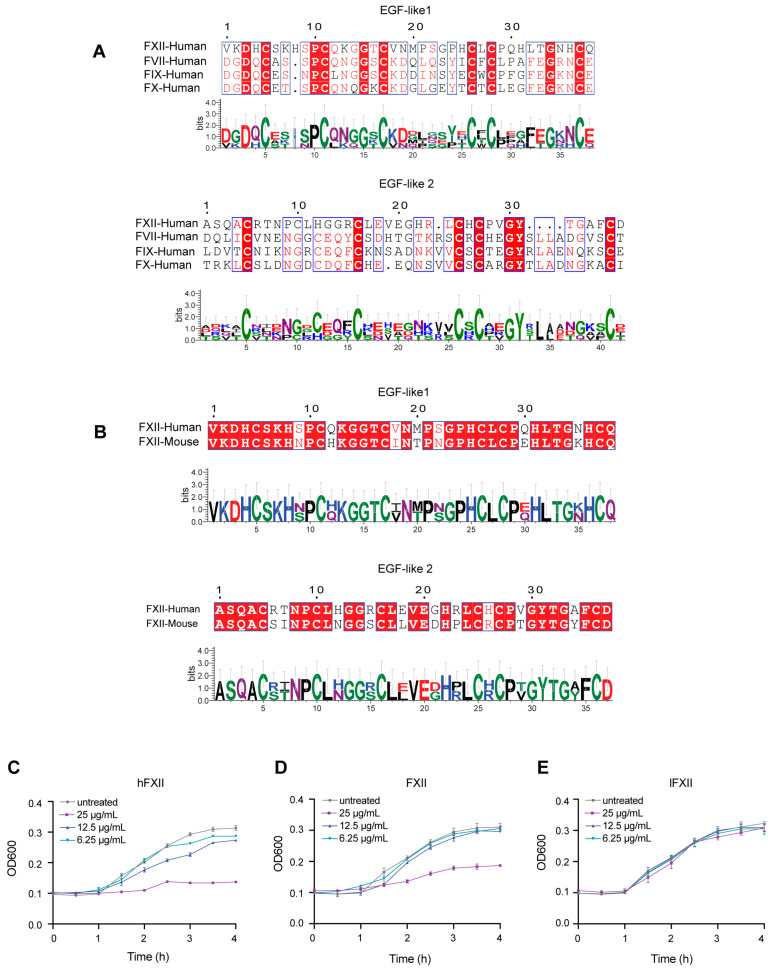
FXII exhibits antibacterial activity in vitro. (**A**,**B**) Conservative analysis of the amino acid sequences of the EGF-like domain. (**A**) The amino acid sequence similarity among FXII, FVII, FIX, and FX. Alignment of the EGF-like1 and EGF-like2 domains of human FXII with those of FVII, FIX, and FX. (**B**) Alignment of the EGF-like1 and EGF-like2 domains between human and mouse FXII. The colors reflect the similarity (red boxes and white characters for conserved residues; red characters for similarity in a group; blue frames for similarity across groups. (**C**–**E**) Determination of the antibacterial curve of recombinant proteins against *P. aeruginosa*. Growth kinetic curves of *P. aeruginosa* at different concentrations of hFXII, FXII, or lFXII (6.25, 12.5, and 25 μg/kg). Amino acid alignment figure was drawn using ESPript 3.0 and WebLogo 3.7.9. All data are shown as mean ± SD. N = 3.

As the activator of the intrinsic coagulation pathway, FXII is traditionally regarded as non-essential for hemostasis but is associated with pathological thrombosis [31,32]. Previous studies of our lab revealed that FVII, FIX, and FX possess antibacterial activity, with the minimal inhibitory structural domain identified as the EGF-like domains. Since FXII contains two EGF-like domains in its heavy chain, we hypothesized that FXII might share similar antibacterial properties. To investigate the potential antibacterial function of FXII, we analyzed the amino acid sequence similarity between FXII and FVII, FIX, and FX. The alignment of the EGF-like1 and EGF-like2 domains of human FXII with those of FVII, FIX, and FX revealed high sequence similarity and conserved homology, suggesting the functional conservation of the EGF-like domains across these proteins (Figure 1A). Mouse FXII protein exhibits high homology and functional conservation with human FXII regarding amino acid sequence (Figure 1B), domain composition, activation pathway, and downstream substrates. For these reasons, murine FXII was utilized in this study to explore its pathophysiological relevance and the essential role in organisms.

To validate the antibacterial activity of FXII and elucidate its mechanism, we constructed three recombinant plasmids that express full-length FXII (FXII), the heavy chain (hFXII), and the light chain (lFXII) of FXII (Figure S1A). After incubating FXII, hFXII, and lFXII with *P. aeruginosa*, the growth kinetic measurements showed that FXII and hFXII significantly inhibited bacterial growth at 25 μg/mL. In contrast, lFXII did not exhibit any inhibitory effect (Figure 1C–E). These results demonstrate that FXII has an antibacterial function through the heavy chain. We further evaluated the minimum bactericidal concentrations (MBCs) of hFXII and FXII against clinically relevant multidrug-resistant Gram-negative pathogens, *A. baumannii* and *P. aeruginosa*. For *A. baumannii*, the MBCs of hFXII and FXII were 17.89 ± 0.27 μg/mL and 24.23 ± 1.73 μg/mL, respectively. For *P. aeruginosa*, the MBCs were 18.72 ± 0.34 μg/mL (hFXII) and 18.80 ± 1.11 μg/mL (FXII) (Table S1). These results demonstrate the potent bactericidal effects of hFXII and FXII against multidrug-resistant strains in vitro.

To explore whether the antibacterial mechanism of FXII parallels that of FVII, using LPS hydrolysis to inhibit the growth of Gram-negative bacteria, we incubated *P. aeruginosa* LPS with hFXII or lFXII. hFXII hydrolyzed LPS in a concentration-dependent manner, with complete degradation observed at 1 μg of protein (Figure S1B). In contrast, lFXII, TBS, and gradient concentrations of BSA failed to hydrolyze LPS. Through in vitro antibacterial experiments, we demonstrated that FXII exerts its antibacterial effects by hydrolyzing LPS, the major component of the Gram-negative bacterial cell wall envelope, via its heavy chain that contains EGF-like domains.

### 2.2. FXII Deficiency Impairs Antibacterial Capacity

To investigate the pathophysiological role of FXII in vivo, we generated FXII-deficient mice using the Cre-loxP system [33]. For FXII^−/−^ mice, we assessed the knockout efficiency by detecting liver DNA, RNA, and plasma protein of FXII. Liver DNA analysis utilizing PCR with primers P5/P6 (557 bp product for recombined alleles) and P5/P7 (858 bp product for wild-type alleles) confirmed successful FXII gene deletion in tamoxifen-treated mice (FXII^−/−^). Meanwhile, corn oil-treated controls (FXII^f/f^) retained wild-type alleles (Figure S2A–C). QPCR revealed a significant reduction in liver FXII mRNA expression in FXII^−/−^ mice compared to controls (Figure S2D). Western blot analysis showed a 70.8 ± 7.6% decrease in plasma FXII protein levels (Figure S2E). As the initiator of the intrinsic coagulation pathway, FXII deficiency specifically prolonged the activated partial thromboplastin time (aPTT) [34] without affecting the prothrombin time (PT), fibrinogen (FIB), and thrombin time (TT) (Figure S2F–I). Reconstitution of FXII^−/−^ plasma with recombinant FXII (0–10 μg/mL) enabled restoration in a dose-dependent manner, achieving wild-type levels at 10 μg/mL (Figure S2J). We successfully constructed a mouse model with FXII deficiency and effectively restored it. Plasma from FXII^f/f^ mice led to bacterial death when incubated with *P. aeruginosa*, whereas FXII^−/−^ plasma lacked this activity (Figure S3A). This directly confirms the bactericidal role of FXII in plasma.

**Figure 2:**
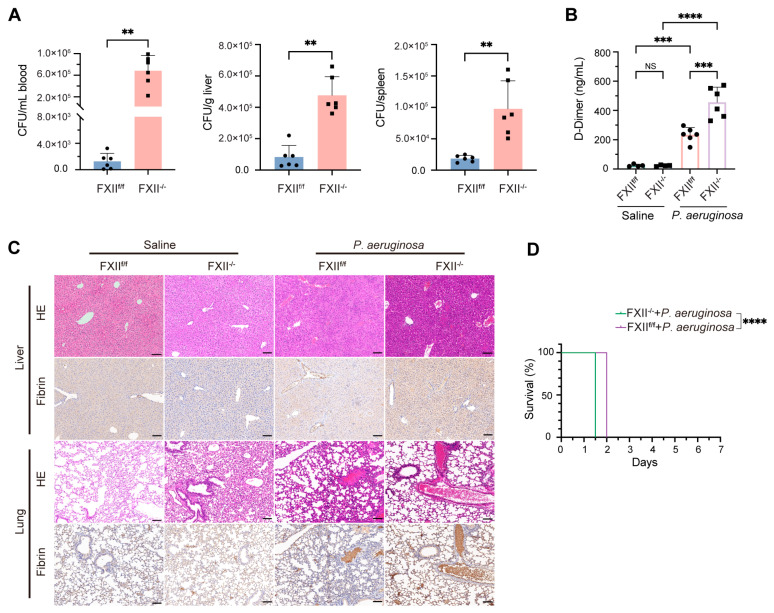
FXII deficiency impairs antibacterial capacity. (**A**) Quantifying bacterial load in the blood, liver, and spleen of FXII^−/−^ and FXII^f/f^ mice after *P. aeruginosa* (1.2 × 10^8^ CFU) was challenged for 20 h via tail intravenous injection. (**B**,**C**) FXII^−/−^ and FXII^f/f^ mice were subjected to *P. aeruginosa* (1.2 × 10^8^ CFU) or saline for 20 h. (B) ELISA detected the plasma level of D-dimer. (**C**) Representative images of HE and IHC staining of fibrin in livers and lungs. scale bar: 100 μm. (**D**) Kaplan–Meier survival plots for FXII^f/f^ vs. FXII^−/−^ mice treated with *P. aeruginosa* (1.6 × 10^8^ CFU) (*n* = 8 to 10 mice per group). Data are shown as mean ± SD. ** *p* < 0.01; *** *p* < 0.001; **** *p* < 0.0001. N = 4 to 6 mice per group. Black scale bar 100 μm. NS, not significant.

To explore whether FXII has a universal antibacterial effect, we constructed mouse models of *P. aeruginosa* infection through intraperitoneal and tail vein injection at a dose of 6 × 10^7^ CFU. FXII^−/−^ mice showed significantly higher bacterial loads in peritoneal fluid, blood, liver, and spleen than FXII^f/f^ mice in two infection models (Figure S3B,C). These results demonstrated impaired antibacterial activity in FXII-deficient mice. We examined the in vivo antibacterial activity of FXII. To further discuss the influence of the antibacterial function exerted by FXII on coagulation indices in mice, we established a high-dose *P. aeruginosa* (1.2 × 10^8^ CFU) infection model. FXII^f/f^ mice exhibited excellent antibacterial activity (Figure 2A). D-dimer serves as a valuable marker of coagulation and fibrinolysis activation [35]. FXII^f/f^ exhibited lower D-dimer levels after infection with *P. aeruginosa* than FXII^−/−^ mice (Figure 2B). We proposed that this occurrence might be due to the direct bactericidal activity of FXII, leading to reduced activation of the coagulation system by *P. aeruginosa*. Accordingly, less fibrin deposition in the liver and lungs was also revealed by immunohistochemical (IHC) staining. It effectively lowers the tendency to form fibrin (Figure 2C). Additionally, a deficiency of FXII shortens the survival time of mice to some extent (Figure 2D). The antibacterial experiment in FXII^−/−^ mice indicates that FXII has a practical antibacterial function in mice through direct antibacterial action. FXII deficiency exacerbates bacterial dissemination in infection models.

### 2.3. Recombinant Proteins Restore Antibacterial Capacity Impaired by FXII Deficiency

**Figure 3:**
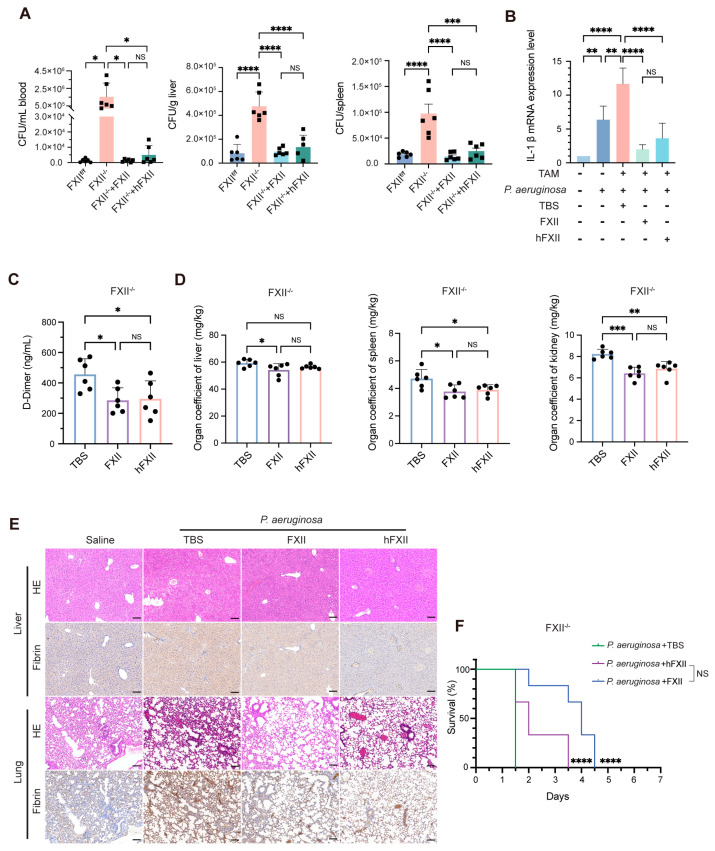
Recombinant proteins restore antibacterial capacity impaired by FXII deficiency. (**A**,**B**) FXII^f/f^ and FXII^−/−^ mice were subjected to *P. aeruginosa* (1.2 × 10^8^ CFU), and then the FXII^−/−^ group was primed with TBS, FXII, or hFXII (125 μg/kg) via tail intravenous injection separately for 20 h. (**A**) Quantifying bacterial load in the blood, liver, and spleen. (**B**) The IL-1β mRNA level of the liver was detected using qPCR. (**C**–**E**) FXII^−/−^ mice were subjected to *P. aeruginosa* (1.2 × 10^8^ CFU) plus TBS, FXII, or hFXII (125 μg/kg) via tail intravenous injection separately for 20 h. (**C**) ELISA detected the plasma level of D-dimer. (**D**) Organ coefficient of liver, spleen, and kidney. (**E**) Representative images of HE and IHC staining of fibrin in livers and lungs. (**F**) Kaplan–Meier survival plots for FXII^−/−^ mice treated with *P. aeruginosa* (1.6 × 10^8^ CFU) and then primed with FXII or hFXII (125 μg/kg), TBS as control, via tail intravenous injection (*n* = 8 to 10 mice per group). Data are shown as mean ± SD. * *p* < 0.05; ** *p* < 0.01; *** *p* < 0.001; **** *p* < 0.0001. N = 4 to 6 mice per group. NS, not significant.

To further explore the role of different FXII domains in response to bacterial infection in mice, we investigated the effects of restoring antibacterial and coagulation functions in *P. aeruginosa*-infected FXII^−/−^ mice via tail intravenous infection. Subsequently, FXII and hFXII proteins were introduced in the same manner to study the mechanism behind the antibacterial activity of FXII. Both FXII and hFXII significantly reduced bacterial loads in the blood, liver, and spleen (Figure 3A), demonstrating the effective restoration of antibacterial function in FXII^−/−^ mice. Compared to FXII^f/f^, FXII^−/−^ mice exhibited markedly higher levels of IL-1β mRNA after infection. Treatment with recombinant FXII or hFXII significantly lowered IL-1β levels in FXII^−/−^ mice, showing no significant difference between the two proteins (Figure 3B). At the same time, both proteins reduced plasma D-dimer levels (Figure 3C) and restored organ coefficient (Figure 3D) in infected FXII^−/−^ mice.

HE and IHC revealed that *P. aeruginosa* induced liver and lung damage in FXII^−/−^ mice, characterized by vascular congestion, hepatocyte edema, inflammatory infiltration, and fibrin deposition. The administration of FXII or hFXII alleviated multiple pathological indicators (Figure 3E). Both FXII and hFXII significantly prolonged the survival time of FXII^−/−^ mice infected with a lethal dose of *P. aeruginosa*, with no significant difference observed between the two treatments (Figure 3F). These findings confirm that recombinant FXII and hFXII exert comparable antibacterial effects in vivo, restoring host defense and mitigating infection-driven mortality.

### 2.4. Antibacterial Role of FXII in a DIC Model

Disseminated intravascular coagulation, a life-threatening complication frequently associated with sepsis, is characterized by widespread microvascular thrombosis. It is strongly associated with systemic microthrombosis and correlates with high mortality rates [11,36]. Nevertheless, the molecular mechanisms underlying the progression from bacterial infections to DIC remain incompletely understood. To elucidate the pathophysiological role of FXII in DIC pathogenesis, we developed a *P. aeruginosa*-induced DIC model to explore how FXII mediates the interplay between its dual functions in host antibacterial defense and coagulation activation. In the *P. aeruginosa*-induced DIC model, plasma FXII levels significantly decreased after infection (Figure S4A), and aPTT was also prolonged (Figure S4B), reflecting excessive consumption due to hyperactivation of the intrinsic coagulation pathway. Simultaneously, coagulation assays revealed elevated fibrinogen (FIB), while PT and TT were unaffected (Figure S4B). Platelet and white blood cell (WBC) counts dropped sharply (Figure S4C,D), confirming successful DIC modeling.

**Figure 4:**
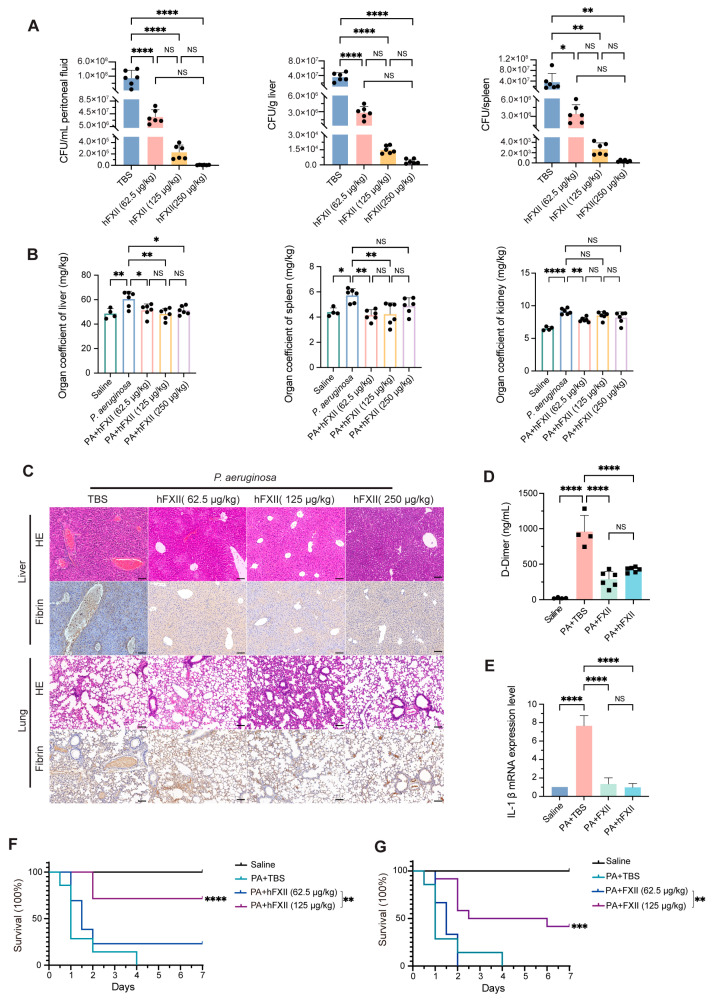
Antibacterial role of FXII in a DIC model. (**A**–**C**) Wt mice were primed with *P. aeruginosa* with the dose of 1 × 10^7^ CFU and 2.25 × 10^8^ CFU at 6 h intervals and then primed with hFXII (0, 62.5, 125, or 250 μg/kg) for 20 h. (**A**) Quantification of bacterial load in peritoneal fluid, liver, and spleen. (**B**) Organ coefficient of liver, spleen, and kidney. (**C**) Representative images of HE and IHC staining of fibrin in livers and lungs. (**D**,**E**) *P. aeruginosa*-induced DIC mice primed with FXII or hFXII (125 μg/kg). (**D**) The plasma level of D-dimer was detected using ELISA. (**E**) IL-1β mRNA levels of the liver were detected using qPCR. (**F**,**G**) Wt mice were primed with *P. aeruginosa* with the dose of 1 × 10^7^ CFU and 2.5 × 10^8^ CFU at 6 h intervals and then primed with hFXII or FXII (0, 62.5, and 125 μg/kg). Kaplan–Meier survival plots for hFXII (**F**) or FXII (**G**) treatment (*n* = 12 to 14 mice per group). PA: *P. aeruginosa*. Data are shown as mean ± SD. * *p* < 0.05; ** *p* < 0.01; *** *p* < 0.001; **** *p* < 0.0001. N = 4 to 6 mice per group. NS, not significant.

Recombinant FXII and hFXII mitigated DIC progression. FXII and hFXII (62.5, 125, 250 μg/kg) effectively decreased bacterial loads in the peritoneal fluid, liver, and spleen (Figure 4A, Figure S4E–G). hFXII, which lacks coagulation activity, diminished organ injury in the liver, spleen, and kidney (Figure 4B). However, FXII has a comparatively minor impact on reducing organ damage (Figure S4H). We assume that hFXII plays an antibacterial role but does not activate the intrinsic coagulation pathway. Histopathological analysis revealed severe hepatic and pulmonary damage in DIC mice, including vascular congestion, fibrin deposition, and inflammatory infiltration. Both FXII and hFXII reduced fibrin accumulation and inflammation, with hFXII demonstrating superior efficacy in minimizing hepatic injury (Figure 4C, Figure S4I). FXII and hFXII significantly lowered D-dimer concentrations (Figure 4D) and IL-1β mRNA levels (Figure 4E), lowering thrombotic risk and inflammation. Survival studies indicated that the high dose of FXII (125 μg/kg) achieved a survival rate of 41.7% (Figure 4F), and hFXII significantly improved survival to 71.4% (Figure 4G) in DIC.

The results demonstrated that both FXII and hFXII effectively diminished the progression of DIC through their antibacterial functions, providing novel insights into the pathogenesis of DIC and other infection-associated thrombotic diseases. Notably, FXII directly regulates bacterial activity and coagulation cascades, thereby critically influencing disease outcomes. Furthermore, FXII may serve as a promising therapeutic target to ameliorate thrombosis by balancing its dual roles in antibacterial defense and coagulation activation without relying on conventional anticoagulants. This discovery advances our understanding of the intricate interplay between inflammatory and coagulatory mechanisms.

## 3. Discussion

FXII, the initiator of the intrinsic coagulation pathway, is traditionally considered non-essential for hemostasis but is associated with innate immunity [37,38]. Many efforts have been made to determine how FXII contributes to innate immunity by activating the contact system, thus driving the immune response. The activation of FXII further stimulates the plasma contact system, promoting pro-inflammatory and pro-coagulant pathways, the complement system, and the fibrinolytic pathway. The significance of FXII may have been underestimated.

Based on earlier findings from our lab, FVII, FIX, and FX exhibit antibacterial activity through their light chains, which contain EGF-like domains. Further studies on FVII revealed that the EGF-like domains are a minimal functional domain, which exerts antibacterial activity by hydrolyzing LPS in Gram-negative bacteria [30]. We investigated whether FXII, which harbors two conserved EGF-like domains in its heavy chain, shares similar antibacterial properties. This could help us identify the proper role of FXII. Sequence alignment of the EGF-like1 and EGF-like2 domains across FVII, FIX, FX, and FXII revealed high conservation. We verified that FXII and hFXII, but not lFXII, significantly inhibited the growth of *P. aeruginosa*. Additionally, hFXII hydrolyzed the LPS concentration-dependently, demonstrating its antibacterial mechanism. EGF-like domains are widely distributed in proteins from diverse species [39,40]. Our studies confirmed the antibacterial activity in FVII, FIX, FX, and FXII. Further investigation is necessary to determine whether this antibacterial function extends to other proteins containing EGF-like domains. Moreover, systematic research is required to investigate the distribution of EGF-like domain-containing proteins across various species, evaluating their role in biological evolution and the evolutionary direction of their antibacterial function.

FXII^−/−^ mice were generated using the Cre-loxP system to explore the role of FXII. Plasma from FXII^−/−^ mice lacked bactericidal activity against *P. aeruginosa*, confirming that natural FXII participates in killing bacteria. In infection models, FXII^−/−^ mice showed higher D-dimer levels. This demonstrates that FXII is targeted in killing bacteria in vivo, reducing *P. aeruginosa* activation of the coagulation pathway and thrombin formation. FXII^−/−^ mice treated with recombinant FXII or hFXII restored their antibacterial capacity, reducing the risk of thrombosis. No significant difference in antibacterial activity was observed between the FXII and hFXII treatment groups, given that hFXII lacks coagulation activity. This indicates that the protein has direct antibacterial effects, suggesting that the antibacterial function is not mediated by indirect activation of contact and complement systems. In DIC models, hFXII outperformed FXII in mitigating hepatic injury, underscoring its therapeutic potential. Furthermore, hFXII significantly improved survival to 71.4% compared to 41.7% for FXII, emphasizing the benefit of separating antibacterial and procoagulant functions while offering a safer therapeutic strategy. Our findings redefine FXII as a dual-function protein that serves as a bifunctional nexus bridging coagulation and antibacterial defense, offering novel insights into its pathophysiological duality.

WHO reports *P. aeruginosa* and *A. baumannii* as key pathogens due to their antibiotic resistance, posing a serious threat to global health [18]. Our study proposes FXII as a potential novel antibacterial target. By balancing antibacterial and anticoagulant effects, hFXII may directly eliminate bacterial pathogens while mitigating infection-driven thrombosis—a strategy addressing the clinical challenge of managing infection-induced DIC without exacerbating coagulopathy. This study establishes the direct antibacterial role of FXII; however, the interplay between its coagulation and immune functions warrants further exploration. We propose that the intrinsic coagulation pathway is an innate immune system, where the dual function of FXII plays an immune role. On one hand, it drives coagulation to trap bacteria by forming thrombin [41]; on the other hand, FXII directly kills bacteria. The balance between the two functions is crucial to make this work efficiently. When it is activated excessively, it leads to thrombosis.

This study redefines the immune landscape by revealing previously unrecognized antibacterial activity of FXII, offering transformative insights into the pathogenesis of infection-associated thrombotic disease. Specifically, the equilibrium between antibacterial and procoagulant activities of FXII in Gram-negative bacterial infections may dictate disease progression. These findings not only illuminate the pivotal role of the intrinsic coagulation pathway in immune defense but also emphasize its pathophysiological significance in severe infectious diseases, such as bacterial intra-abdominal infections, bloodstream infections, and disseminated intravascular coagulation. This study deepens new avenues for developing FXII dural function against severe infection-related diseases.

## 4. Materials and Methods

### 4.1. Cell Lines and Bacterial Strain

CHO-K1 (NCACC, Shanghai, China) cells were cultured in Ham’s F12-K (Gibco, Karlovy Vary, CA, USA) supplemented with 10% fetal bovine serum (FBS) (ExCell Bio, Suzhou, China) and 1% penicillin–streptomycin (Hyclone, Logan, UT, USA) at 37 °C in a 5% CO_2_ incubator. The cell lines were mycoplasma-free and passaged no more than 4 weeks after resuscitation.

*P. aeruginosa* and *A. baumannii* were donated by the First Affiliated Hospital of Chengdu Medical College, Chengdu, Sichuan, China.

### 4.2. Mice

The mice involved in the experiment were the C57BL/6J background strain. Importantly, 6–8-week-old (20–22 g) male mice were used in this study. All experimental animals were housed in an Individually Ventilated Cage (IVC) system. The environmental parameters were controlled at a temperature of (25 ± 1 °C), a relative humidity of (55 ± 15%), and a 12 h light–dark cycle with rhythmic lighting conditions. All experimental mice were mated and produced under specific pathogen-free (SPF) conditions.

The FXII-eCKO1 mice were constructed by the Shanghai Model Organisms Center (Shanghai, China). The C57BL/6-*Gt(ROSA)26or^tm6(CAG-iCre/ERT2)^*/Bcgen mice were purchased from Biocytogen (Nantong, China). The floxed mice were mated with B-CAG-iCreERT2 (Cre^+^) mice to generate FXII^f/f^Cre^+/−^ mice, which were used to create *FXII* gene knockout mice. Transgenic mice used for experiments were confirmed to be the desired genotype through polymerase chain reaction (PCR) analysis of tail DNA. The sequence of the PCR primers is as follows:

FXII-P3: 5′-ACTGTTGGGCGCTGGTTGG-3′;
FXII-P4: 5′-GCTGGATTTGGTCATGGTGTTT-3′;
Cre-Mut-F: 5′-TGCGGGCTCTACTTCATCG-3′;
Cre-Mut-R: 5′-GAGGGGGAAAGCGAAAGTCC-3′;
Cre-Wt-F: 5′-AGTCGCTCTGAGTTGTTATCAG-3′;
Cre-Wt-R: 5′-TGAGCATGTCTTTAATCTACCTCGATG-3′.

Tamoxifen (100 mg/kg, Selleck, Houston, TX, USA, Cat# S1238) dissolved in corn oil (aladdin, Shanghai, China, Cat# C116023) was administered to FXII^f/f^Cre^+/−^ mice via intraperitoneal for five consecutive days to obtain FXII^−/−^ mice, and the control group received only corn oil (FXII^f/f^). There is a one-week waiting period between the final injection and the experiment. Following DNA extraction from the liver (TIANGEN, Beijing, China, Cat# DP304-02), PCR was employed to determine whether the FXII had been deleted. Liver RNA was used for quantitative real-time PCR to detect FXII transcription levels. Mouse plasma was obtained, and knockdown efficiency was assessed using Western blot. FXII^−/−^ mice were verified via PCR using the primers FXII-P5: 5′-GCCACCTTTTGACAGTGATGAG-3′; FXII-P6: 5′-AGCTTCTCCACAGCCACAAT-3′; and FXII-P7: 5′-TGATGGATGCTACCAAACTGGA-3′. At the end of the study, mice that were still alive were euthanized via CO_2_ exposure. All experiments were approved by the Animal Ethics Board and Animal Care and Use Committee at the School of Life Sciences, Sichuan University, and efforts were made to minimize suffering.

### 4.3. Protein Production

The vector pcDNA3.1(+) was used to construct the expression plasmids pcDNA3.1-FXII-His, pcDNA3.1-hFXII-His, and pcDNA3.1-lFXII-His. Murine Immunoglobulin Kappa (Ig*κ*) replaced the original secretion signal peptide of mouse FVII (Ori). All plasmids were confirmed through DNA sequencing. For stable eukaryotic expression of His-tagged FXII, hFXII, and lFXII, CHO-K1 cells were transfected with the plasmids pcDNA3.1-FXII-His, pcDNA3.1-hFXII-His, and pcDNA3.1-lFXII-His, respectively. The high-expression clones were screened using Geneticin (G418) (Selleck, Houston, TX, USA). The selected high-expression sub-clones were chosen for subsequent protein expression and purification. HisPur^TM^ Cobalt Resin (Thermo Fisher Scientific, Waltham, MA, USA) was employed to purify His-tagged proteins. To equilibrate the resin, we used an equilibration buffer (50 mM sodium phosphate, 300 mM sodium chloride, 10 mM imidazole, pH 7.4). Protein binding was treated using wash buffer (50 mM sodium phosphate, 300 mM sodium chloride, 10 mM imidazole, pH 7.4), followed by the application of elution buffer (50 mM sodium phosphate, 300 mM sodium chloride, 150 mM imidazole, pH 7.4). The purified proteins were dialyzed against dialysis buffer (150 mM NaCl, 25 mM Tris-HCl, pH 7.4). The entire purification process was maintained at a temperature of 4 °C.

### 4.4. Growth Kinetics Analysis

*P. aeruginosa* was cultivated in Luria–Bertani (LB) medium at 37 °C until the OD600 reached 0.6, then diluted 10-fold with fresh medium. Samples of 100 μL from the dilutions were added to each well of microtiter plates containing pre-warmed LB supplemented with FXII, hFXII, lFXII, or no drug (untreated). The plates were covered and incubated at 37 °C with shaking at 185 rpm for various incubation periods. The OD600 measurements were performed using a universal microplate spectrophotometer (BioTek, Winooski, VT, USA). LPS (100 μg/mL) was mixed with hFXII or lFXII in Tris-buffered saline (TBS, pH 7.4), and the resulting mixtures were incubated at 37 °C with shaking at 185 rpm.

### 4.5. Analysis of LPS Degradation by Electrophoresis

*P. aeruginosa* LPS (500 ng), purified using a method of hot aqueous-phenol extraction [42,43], was mixed with various concentrations (0.125, 0.25, 0.5, and 1 μg) of hFXII, lFXII, or BSA, with TBS serving as a negative control. The resulting mixtures were incubated at 37 °C while shaking at 160 rpm. After incubation, the reaction mixtures were collected by centrifugation at 17,000× *g* and subsequently subjected to SDS-PAGE analysis and staining with silver [44]. The gel was rinsed three times with double-distilled water (ddH_2_O), with 5 min for each rinse. LPS was oxidized in the gel with 30% ethanol, 10% acetic acid, and 7 g/L periodic acid at room temperature for 30 min. The gel was then washed three times with ddH_2_O for 5 min. It was stained for 30 min with freshly prepared staining solution (1 g/L of silver nitrate) at 30 °C, and then washed once with ddH_2_O for 10 s. The color was developed by reducing 30 g/L of pre-chilled sodium carbonate and 0.02% formaldehyde (added immediately before use). The gel color was halted via exposure to 10% acetic acid, followed by re-equilibration washes in ddH_2_O. Finally, it was preserved in 70% acetic acid [45].

### 4.6. Western Blot and ELISA

The plasma samples were fractionated using SDS-PAGE and transferred electronically onto PVDF membranes at 100 V for 1.5 h at 4 °C. The membranes were then rinsed with PBST buffer (0.2% Tween-20 in phosphate-buffered saline), blocked for 1 h in PBST containing 5% non-fat milk powder, and incubated overnight at 4 °C with mouse coagulation Factor XII antibody (Proteintech, Wuhan, China). The membranes were washed with PBST before incubating with horseradish peroxidase (HRP)-conjugated IgG (Proteintech, Wuhan, China) for 1 h. The resulting blots were visualized using the ShinEasy Subpico ECL kit (Foregene, Chengdu, China) and the Bio-Rad (Hercules, CA, USA) ChemiDoc Touch Imaging System.

### 4.7. Quantitative Real-Time Polymerase Chain Reaction

Quantitative real-time polymerase chain reaction (qPCR) was conducted using a StepOnePlus^TM^ system (Applied Biosystems, Carlsbad, CA, USA). The expression levels of FXII and IL-1β mRNA were assessed via qPCR. RNA was isolated using the AG RNAex Pro Reagent (Accurate Biology, Changsha, China, Cat# AG21101) and reverse-transcribed to cDNA with RT Easy^TM^ I (Foregene, Chengdu, China, Cat# RT-01011). Real-time fluorescent quantitative PCR was performed in StepOnePlus^TM^ (Applied Biosystems, Foster, CA, USA). Genes were amplified for melting curves using Real Time PCR Easy^TM^-SYBR Green I (Foregene, Chengdu, China, Cat# QP-01011). The mRNA expression was normalized to GAPDH. The FXII primers were as follows:

FXII-F: 5′-AATCCGTGCCTTAATGGGGG-3′,
FXII-R: 5′-TCATAGCAGGTCGCCCAAAG-3′;
GAPDH-F: 5′-AGGTCGGTGTGAACGGATTTG-3′,
GAPDH-R: 5′-TGTAGACCATGTAGTTGAGGTCA-3′;
IL-1β-F: 5′-TCAAATCTCGCAGCAGCACATC-3′,
IL-1β-R: 5′-CGTCACACACCAGCAGGTTATC-3′.

### 4.8. Bacterial Load in Blood, Peritoneal Fluid, Liver, and Spleen

The bacterial load in the blood, peritoneal fluid, liver, and spleen of mice was measured as described [46,47]. After 20 h of *P. aeruginosa* inoculation, blood was collected and combined with 109 mmol/L of sodium citrate. Peritoneal fluid was extracted from the peritoneum after saline injection (1 mL), and liver and spleen tissues were obtained from killed mice, weighed, and homogenized in saline. Serial dilutions of blood, peritoneal fluid, and tissue homogenates were inoculated onto LB agar plates and incubated overnight at 37 °C. Colony Forming Unit (CFU) was calculated as CFU/mL for blood or peritoneal fluid, as CFU/g of wet weight for liver, and CFUs for each fresh spleen organ.

### 4.9. Measurement of Coagulation Parameters

The blood samples were promptly combined with sodium citrate anticoagulant and centrifuged at 700× *g* for 15 min at 4 °C, after which the supernatant was collected as the plasma sample for subsequent use. Plasma concentrations of D-dimer were measured with ELISA kits (FineTest, Wuhan, China). Coagulation parameters, including prothrombin time (PT), activated partial thromboplastin time (aPTT), fibrinogen (Fib), and thrombin time (TT), were detected using the Semi-auto Coagulation Analyzer (Wuhan King Diagnostic Technology, Wuhan, China). Mouse whole blood was added to a tube containing ethylenediaminetetraacetic acid (EDTA) disodium salt to detect platelets and white blood cells (WBC) from Wuhan Powerful Biology Technology (Wuhan, China).

### 4.10. Liver and Lung Histology and IHC

The mice were euthanized after 20 h of injection with *P. aeruginosa*. Liver and lung tissues were collected, rinsed with saline, weighed, and preserved in 4% paraformaldehyde. Hematoxylin–eosin staining (HE) and immunohistochemistry (IHC) were performed by Wuhan Powerful Biology Technology (Wuhan, China).

### 4.11. Antibodies and Reagents

Factor XII Monoclonal antibody (66089-1-1g), Transferrin Polyclonal antibody (17435-1-AP), HRP-conjugated Goat Anti-Rabbit IgG(H+L), HRP-conjugated Goat Anti-Mouse IgG(H+L) (SA00001-2), and HRP-conjugated Goat Anti-Mouse IgG(H+L) (SA00001-1) were purchased from Proteintech (Wuhan, China). Mouse FXII ELISA Kit (EM1046) and Mouse D2D ELISA Kit (EM0979) were purchased from FineTest^®^ (Wuhan, China).

HisPur^TM^ Cobalt Resin (89965) was purchased from Thermo Fisher Scientific (Waltham, MA, USA). The Gel & PCR Clean-Up Kit (D2000-01), Gel Extraction Kit (D2500-02), and Endo-Free Plasmid Mini Kit II (D6950-01) were purchased from Omega (Norcross, GA, USA).

Real Time PCR Easy^TM^-SYBR Green I (Foregene, QP-01012). Tamoxifen (C116023) and G418 (S3828) were purchased from Selleck (Houston, TX, USA), and corn oil (D2500-02) was purchased from Aladdin (Shanghai, China).

Fetal Bovine Serum (SH30406.05) and Trypsin (SH40003.01) were purchased from Hyclone (Logan, UT, USA), Ham’s F-12K (L450KJ) was purchased from BasalMedia (Shanghai, China), and LipoMax^®^3000 transfection reagent (HKR3102) was purchased from Bio-Gene (Melbourne, Australia).

Finally, 4% paraformaldehyde (BL539A) was purchased from Biosharp (Beijing, China), and physiological saline solution (M21032906C) was purchased from Sichuan Kelun Pharmaceutical (Chengdu, China).

### 4.12. Statistical Analysis

Statistical analyses were performed using GraphPad Prism 10.1.2 software (GraphPad Software, San Diego, CA, USA). Student’s *t*-test was used to compare differences between the two groups. When more than two groups were compared, one-way analyses of variance (ANOVA) were used. The log-rank (Mantel–Cox) test was conducted to analyze survival curves. All results are expressed as means ± SD, and *p*-values < 0.05 were considered statistically significant.

## Data Availability

Data is contained within the article and Supplementary Material.

## Supplementary Materials

The following supporting information can be downloaded at: https://www.mdpi.com/article/10.3390/ijms26136009/s1.
