# Supplementary material for: Coagulation Factor XII Is an Antibacterial Protein That Acts Against Bacterial Infection via Its Heavy Chain
Source: Int J Mol Sci. 2025 Jun 23;26(13):6009. doi: 10.3390/ijms26136009 (PMC12249503; doi:10.3390/ijms26136009)
Supplement: Supplementary file 1 [file ijms-26-06009-s001.zip › ijms-3688069-supplementary.pdf]

Supplemental Figures and Figure Legends

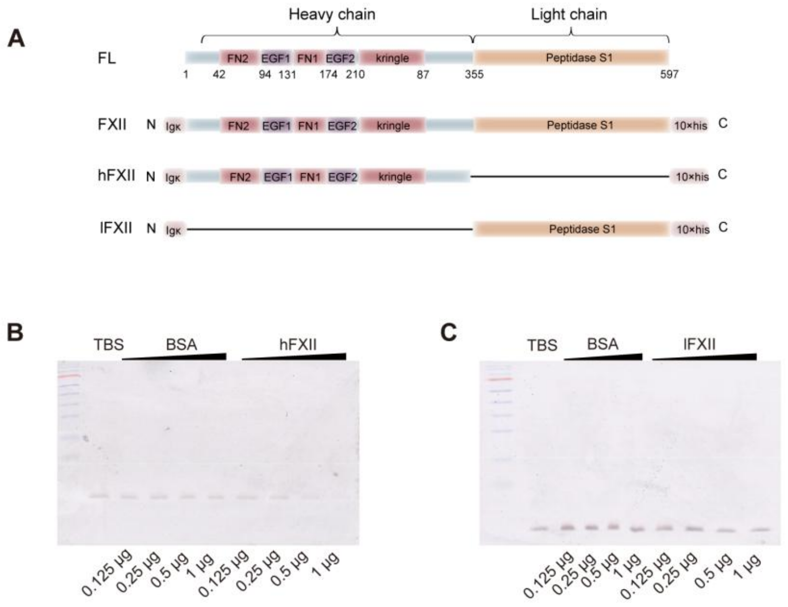

Figure S1. FXII exerts antibacterial activity by hydrolyzing LPS. (A) Schematic representation of domains that mouse FXII, recombinant FXII, hFXII, and lFXII. FL: full length; number: amino acid sites. EGF1: EGF-like1, EGF2: EGF-like2, FN1: fibronectin 1; FN2: fibronectin 2. (B-C) Electrophoretic profiles of LPS hydrolysis. LPS of *P. aeruginosa* (500 ng) incubation with TBS or increased dose of BSA or protein (hFXII and lFXII) separately at 37 ℃ for 3 hours per reaction. Separated LPS samples on SDS-PAGE were examined by silver staining. Data are shown as mean ± SD.

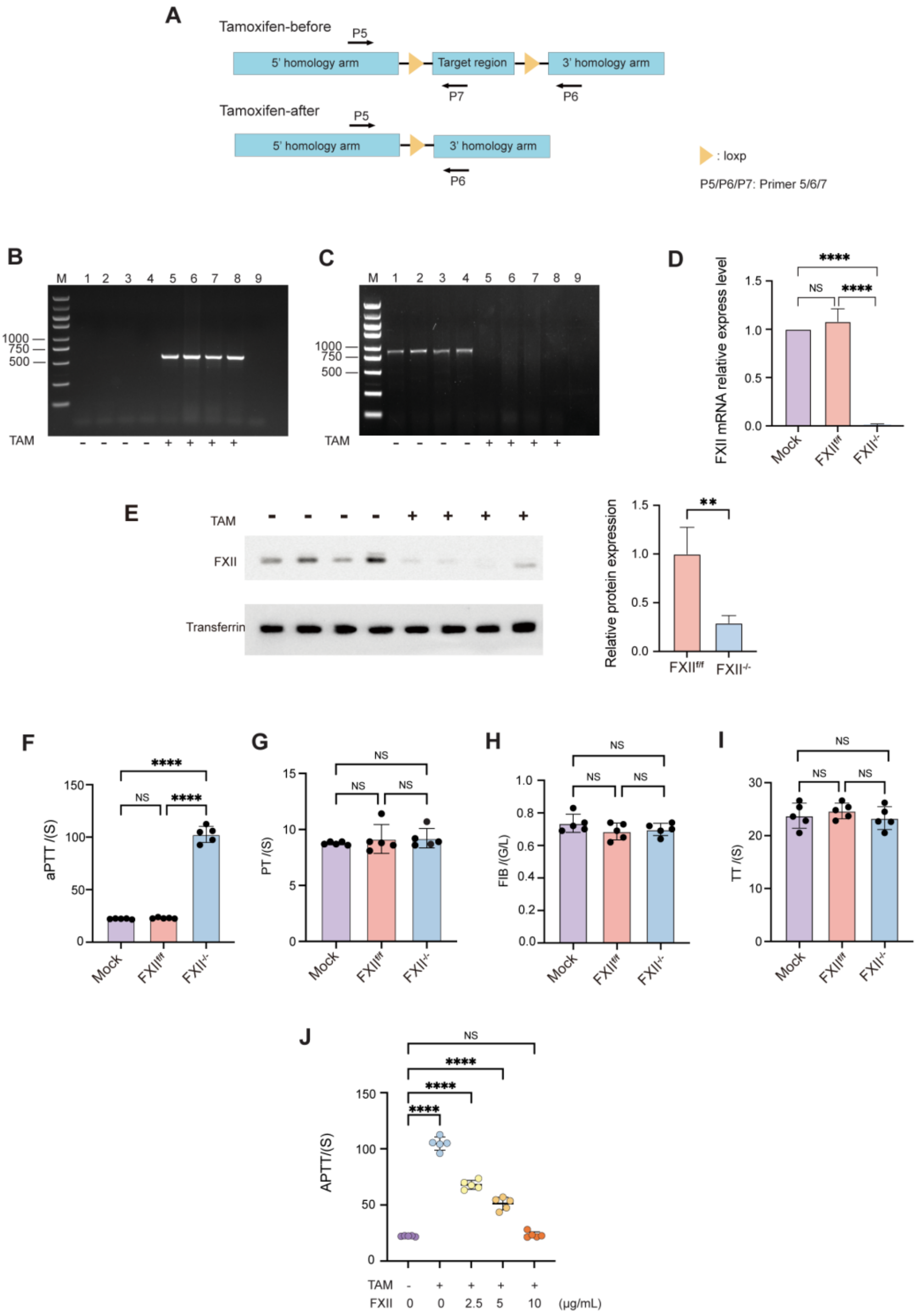

Figure S2. The construction of FXII-eCKO1 conditional knockout mice. (A) Schematic diagram of the Cre activity assay. P5/P6/P7: primer 5/6/7. (B-I) FVII$^{f/f}$Cre$^{+/-}$ mice were primed with tamoxifen (TAM) dissolved in corn oil or an equal volume of corn oil by intraperitoneal injection for five consecutive days. Detection of FXII knockout efficiency after a one-week period following final dosing. The liver of mice was collected for detection using PCR (B-C) and qPCR (D). Liver DNA amplification products generated by primers P5/P6 (B) or P5/P7 (C). Plasma was collected for protein level detection by Western blot (WB) (E) of FXII. (F-G) APTT, PT, FIB, and TT were detected by a semi-automatic hemagglutination instrument. (J) In vitro recovery of FXII$^{-/-}$ plasma. Measurement of aPTT values in plasma from FXII$^{-/-}$ supplemented with

gradient concentrations of FXII (0, 2.5, 5, 10 μg/mL) in vitro, plasma from FXII$^{f/f}$ as control. Data are shown as mean ± SD. ***P*< .01; *****P*< .0001. N = 4 to 5 mice per group. NS, not significant.

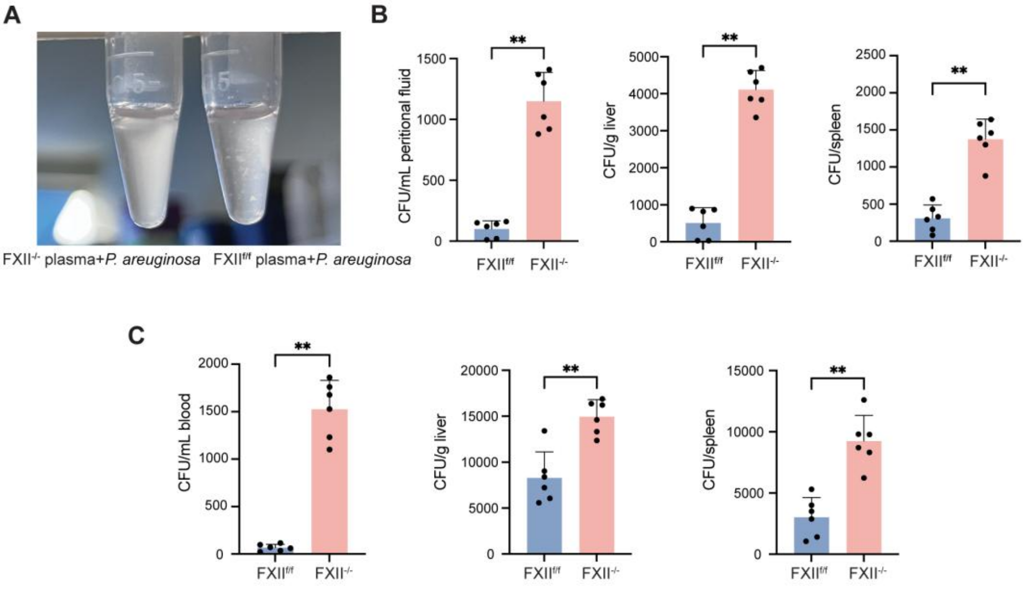

Figure S3. FXII deficiency impairs antibacterial capacity. (A) Incubate equal volumes of *P. aeruginosa* (OD600=0.6) with plasma from FXII$^{-/-}$ or FXII$^{f/f}$ mice at 37°C for 1 hour. (B-C) FXII$^{-/-}$ and FXII$^{f/f}$ mice were subjected to P. aeruginosa (6 × 107 CFU) for 20 hours by intraperitoneal injection (B) or intravenous injection of the tail (C). (B) Quantification of bacterial load in peritoneal fluid, liver, and spleen. (C) Quantification of bacterial load in blood, liver, and spleen. Data are shown as mean ± SD. ***P*< .01. N = 6 mice per group. NS, not significant.

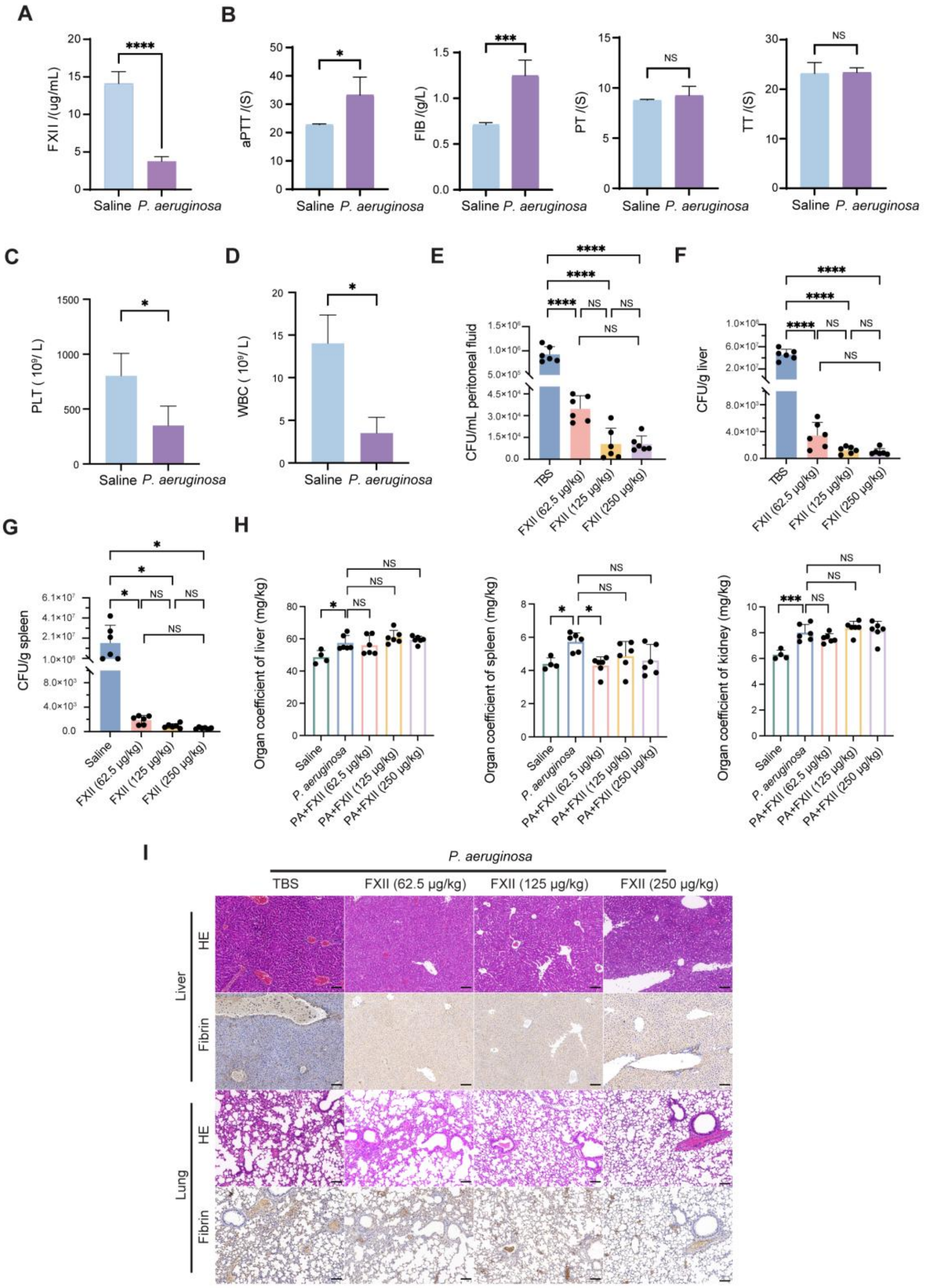

Figure S4. Antibacterial Role of FXII in a DIC Model. (A-D) Wt mice were primed with *P. aeruginosa* with the dose of 1 × 10^7 CFU and 2.25 × 10^8 CFU at 6-hour intervals, and then the detection indicators of the DIC model were examined. (A) FXII plasma levels detected by ELISA. (B) A semi-automatic hemagglutination instrument detected FIB, aPTT, PT, and TT. (C-D) The counts of PLT and WBC. (E-G) Wt mice were primed with *P. aeruginosa* with the dose of 1 × 10^7 CFU and 2.25 × 10^8 CFU at 6-hour intervals and then primed with FXII (0, 62.5, 125, or 250 μg/kg) for 20 hours. Quantification of bacterial load in peritoneal fluid (E), liver (F), and spleen (G). (H) Organ coefficient of liver, spleen, and kidney. (I)

Representative images of HE and IHC staining of fibrin in livers and lungs (100×). Data are shown as mean ± SD. **P*< .05; ***P*< .01; ****P*< .001; *****P*< .0001. N = 4 to 6 mice per group. NS, not significant.

Supplemental Table

Supplemental Table 1. The MBC of hFXII and FXII against *P. aeruginosa* and *A. baumannii.*

Bacteria type	MBC（μg/mL）	
	hFXII	FXII
P. aeruginosa	18.72 ± 0.34	18.80 ± 1.11
A. baumannii	17.89 ± 0.27	24.23 ± 1.73
